# A new phenotype of aldolase a deficiency in a 14 year-old boy with epilepsy and rhabdomyolysis – case report

**DOI:** 10.1186/s13052-022-01228-3

**Published:** 2022-03-04

**Authors:** Lucia Santoro, Dorina Pjetraj, Virtut Velmishi, Carmen Campana, Carlo Catassi, Carlo Dionisi-Vici, Arianna Maiorana

**Affiliations:** 1 Division of Pediatrics, Polytechnic University of Marche, Ospedale Pediatrico “G. Salesi”, Ancona, Italy; 2 Pediatric Service Nr 2 “Mother Teresa” Hospital-Trina, Tirana, Albania; 3 Division of Metabolism, Department of Pediatric Subspecialties, Bambino Gesù Children’s Hospital IRCCS, Rome, Italy

**Keywords:** Glycogen Storage Disease type XII, Aldolase A Deficiency, ALDOA Deficiency, 24 GSD XII, Ketogenic Diet

## Abstract

**Background:**

Glycogen storage disease type XII is a rare metabolic disease resulting from Aldolase A deficiency that causes muscle glycogen accumulation, with crisis of rhabdomyolysis and hemolytic anemia. In the very few cases described, rhabdomyolysis crises are caused by fever and/or exercise and can accompany acute hemolytic anemia. Although currently there is no therapy available for this disease, the guidelines for the management of other forms of glycogen storage diseases recommend a nutritional therapy in order to avoid hypoglycemia or prevent exercise-induced rhabdomyolysis.

**Case presentation:**

In this case report we describe a new phenotype of the disease in a 14-year-old boy, characterized by seizures and rhabdomyolysis. Beside an antiepileptic treatment, we propose a new therapeutic approach based on ketogenic diet in order to supply an energetic substrate for skeletal muscle and neurons.

**Conclusions:**

The anti-epileptic therapy and the dietetic approach were well tolerated by the patient who showed good compliance. This led to a deceleration of the disease with no other acute episodes of seizures and rhabdomyolysis, without any side effects observed.

## Synopsis

A case report of a patient with a new phenotype of Aldolase A deficiency characterized by epilepsy and rhabdomyolysis, Treated with ketogenic diet.

## Background

Glycogen storage disease type XII (GSD XII) is an ultra-rare autosomal recessive disorder caused by aldolase A (ALDOA) deficiency, characterized by hemolytic anemia and rhabdomyolysis with or without myopathy or intellectual disability. Aldolase is a thermolabile glycolytic enzyme responsible for the reversible conversion of fructose-1,6-bisphosphate to glyceraldehyde-3-phosphate and dihydroxyacetone phosphate [1].

Three aldolase isozymes (A, B, and C) are known encoded by 65 different genes. They have different tissue distribution and are differentially expressed during lifetime [2].

Aldolase A is localized in muscle, red blood cells and brain; aldolase B is localized in the liver; while aldolase C occurs in the central nervous system along with aldolase A which is the isoform most abundantly expressed in the human brain [3, 4].

The first case of aldolase A deficiency was reported in 1973 by Beutler et al. [5]. The common phenotype is characterized by rhabdomyolysis and hemolytic anemia triggered by fever or exercise, myopathy, subtle cognitive dysfunction (learning disabilities or delayed language acquisition) [5–9]. No medical therapy has shown to be effective for this rare disease, that is treated only by supportive and symptomatic therapy such as red blood cells transfusions, hydration and rehabilitation.

In this case report we describe a new clinical phenotype of GSD XII characterized by epilepsy and rhabdomyolysis in a 14-year-old boy. We also propose a pharmacological approach in order to prevent epileptic crisis, together with dietetic measures based on ketogenic diet.

## Case presentation

The proband, was a boy aged 14-years when the diagnosis of aldolase deficiency was established. He is the third child of an Albanian couple without consanguinity.

The boy was born after an uneventful pregnancy and delivery, and presented with normal growth and neurologic development. At the age of 2 years he was admitted to the hospital for a severe anemia which required blood transfusion. Unfortunately, no data was available regarding that episode.

At the age of 14 years and 9 months, the child presented a first episode of generalized tonic–clonic seizures associated with loss of consciousness and followed by dark ''coca-cola''–coloured urine. Laboratory examination revealed myoglobinuria with evidence of  hypertransaminasemia, and hyperCPKemia (AST 4559 U/I normal 0–40; ALT 1198 U/l, normal 0–40; CPK 138,000 U/l, normal 0–170). At that time, echocardiography, abdomen ultrasound, brain CT, and EEG were performed at the "Mother Teresa" University Hospital Center in Tirana and no abnormality was detected.

His family history revealed that an older brother, previously diagnosed with epilepsy and rhabdomyolysis at the age of 14 years, died during a sudden status epilepticus at the age of 17 years.

Taking into consideration both clinical and family history, in the suspicion of metabolic disease, exome sequencing was performed and the homozygous pathogenic variant c.1001C > T (p.Ala334Val) of gene ALDOA was localized and classified as likely pathogenic (class 2) according to the recommendation of Centogene (Rostock, Germany) and ACMG (ACMG SF v2.0 2016 update). Polyphen software predicted this change to be as “probably damaging”. On the basis of this finding a diagnosis of GSD XII was done. Segregation studies confirmed the heterozygosity in both parents and in the younger brother.

**Fig. 1:**
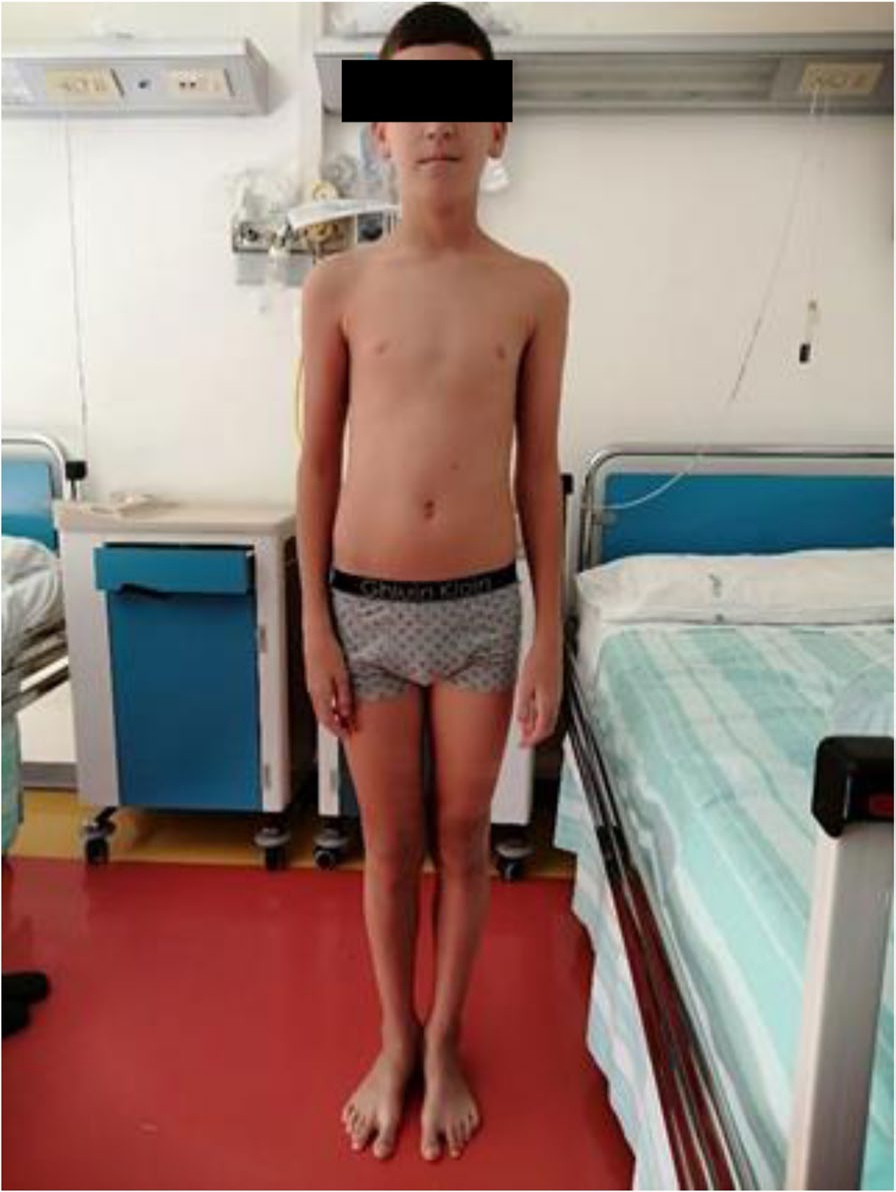
Anterior view: Poor muscle mass in a boy with normal stature

**Fig. 2:**
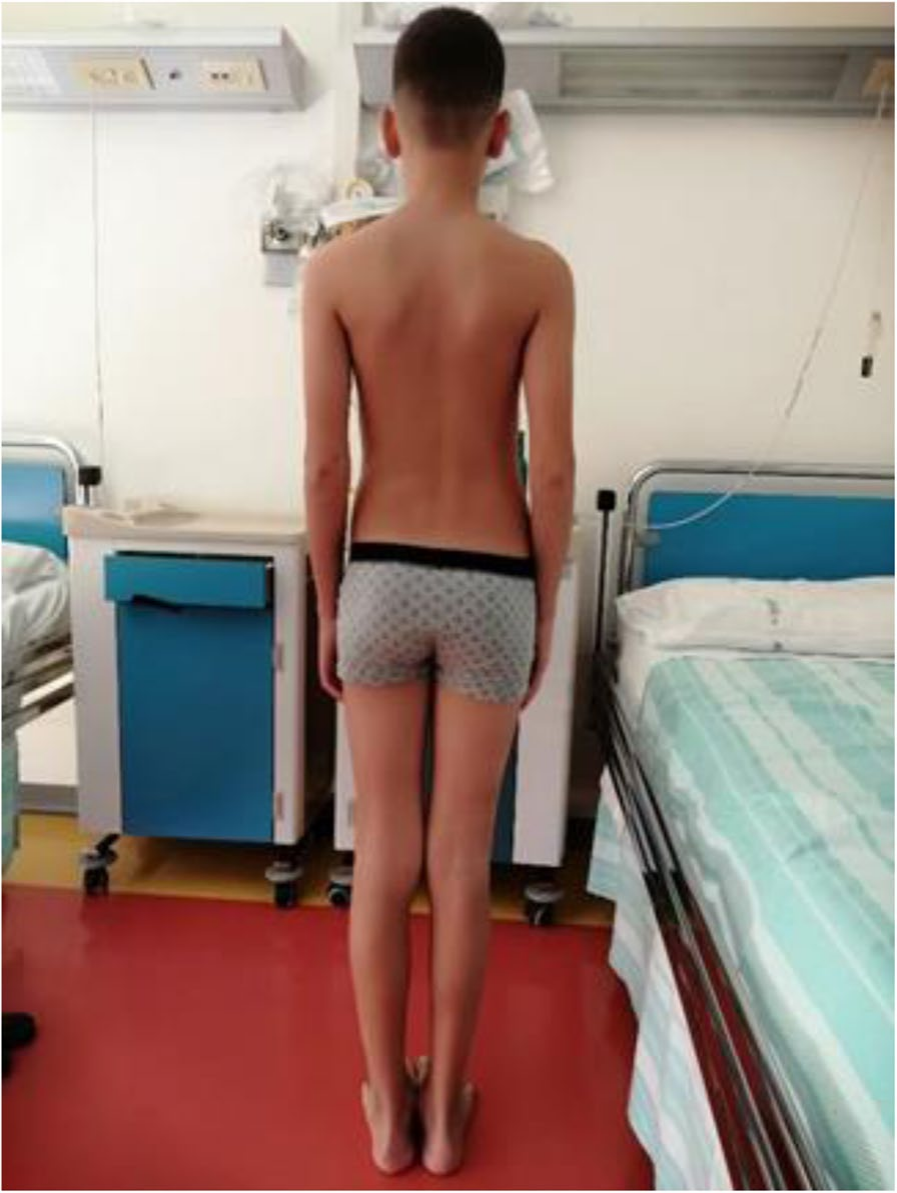
Posterior view: Dorsal-lumbar right convex scoliosis

At the age of 15 years, the boy presented two more critical episodes one month apart with subsequent rhabdomyolysis and myoglobinuria which resolved spontaneously. CPK values were 138,000 U/I and 11,560 U/I respectively. At this time, the patient was referred to the Center for Metabolic Diseases of the Salesi Children's Hospital, Ancona, Italy, for further evaluation. On admission, the child appeared in good general condition. He did not show developmental delays nor appreciable dysmorphic features. He weighed 41 kg (3° p), and was 156 cm tall (90° p). On physical examination, the patient had diminished muscle mass with normal muscle tone and mild dorsal-lumbar right convex scoliosis (Figs. 1 and 2). He did not show any motor limitations, but only mild diffuse myalgia, mainly in the lumbar area. He did not present hepatomegaly. Laboratory examinations revealed the following values: hemoglobin 11.1 g/dl; hematocrit 32.9%; MCV 83 fl, white-cell count 8310/mmc and platelet count 272,000/mmc. The CPK concentration was markedly elevated (14,000 U/l). The levels of the following were also abnormal: AST 465 U/l; ALT 375 U/l; lactate dehydrogenase 542 U/l (normal 0–325). Urinalysis and arterial blood gas analysis were normal. During hospitalization, the child presented two more critical episodes. The first episode occurred in the early morning, during sleep, in apyrexia. He presented with generalized tonic–clonic seizures which resolved after administration of rectal diazepam. Laboratory examination revealed severe metabolic acidosis (Ph 7.2, HCO3- 19 mmol/l, base excess -5.7 mmol/l, anion gap 15 mmol/l), mild renal insufficiency (creatinine level 0.95 mg/dl, normal 0.2–1.3), hyperuricemia (16.8 mg/dl, normal 2.5–7) and hyperCPKemia (33.306 U/l), and hypermyoglobinemia (1148 ng/ml, normal 0–110). Adequate hydration was practiced and rasburicase was prescribed with normalization of urinary biomarkers. An EEG was performed immediately after the crisis and no abnormalities were detected. In contrast, the EEG performed 24 h post-crisis showed sporadic wide slow waves. The second episode, similarly to the first, occurred early in the morning, during sleep. The patient presented metabolic acidosis (ph 7.28, HCO3- 28.7 mmol/l, anion gap 21, EB -7, lactic acid 7.6), hyperuricemia (13.2 mg / dl) and hyperCPKemia (41.870 U/l) and hypermyoglobinemia (1738 ng/ml). As with the previous episode he was treated with adequate hydration and rasburicase therapy.

While immediate resolution of metabolic acidosis and hyperuricemia was achieved, myoglobinuria and high rhabdomyolysis indices persisted, returning to baseline only in the following days (CPK 1612U /l, myoglobin 138 ng/ml). Taking into consideration the patient’s history of early morning seizures, in conjunction with metabolic acidosis, a normocaloric diet was put into place, with particular emphasis placed on the midnight meal consisting of slow-absorbing carbohydrates (cornstarch). This nutritional intervention was introduced alongside an anticonvulsant therapy consisting of Leviteracetam twice daily.

The patient was then referred to the Division of Metabolism at Ospedale Pediatrico Bambino Gesù in Rome for further diagnostic and therapeutic evaluations. The metabolic work-up was always normal, both in the acute phases and in the intervening periods, specifically plasma and urinary amino acids, plasmatic acylcarnitine profile, and urinary organic acids, chromatography of urinary carbohydrates. A 24-h fasting test was performed resulting in normal glycemia, however an elevation of CPK together with ST segment depression at ECG was noticed. After nutrition recovery, both ECG and CPK values normalized. At the same time, the boy presented mild symptoms of pharyngodynia and rhinorrhea and tested positive for Covid-19. In consideration of many studies highlighting the potentially positive effects of ketogenic diet on many neurological diseases and muscular GSD III, V, VII [10–14] ketogenic diet was started to the patient. After five months from the start of ketogenic diet, the patient did not experience any further metabolic decompensations, as reported by phone interviews.

## Materials and methods

Routine hematologic tests, biochemical and genetic analysis, EEG and MRI were performed by standard techniques.

Diet: Induction to the ketogenic regime was started with a ketogenic ratio (fats:proteins plus carbohydrates) of 0.9:1, with progressive increase up to 2.5:1. The macronutrient composition of the 2.5:1 ketogenic diet was 7% proteins (1,1 g/kg), 83% fat and 10% carbohydrates in accordance to the recommended daily energy intake (Total 2900 kcal, 62 kcal/kg/die). The onset of ketogenic diet was controlled by daily capillary blood and urine ketone measurements. For implementing a ketogenic diet, follow-up training and course materials that took into consideration the patient’s Albanian heritage were prepared and country-specific recipes were modified into a ketogenic variant.

Anti-epileptic drugs consisted of Leviteracetam, which was started at the dose of 250 mg bd and progressively increased until 1 gr bd. Drug concentration resulted in a therapeutic range (22.69 μg/mL).

## Discussion and conclusions

Unlike previously described cases, our patient and his brother, who died most likely of the same disease, presented with severe neurological impairment and epileptic seizures during metabolic decompensation. The epileptic seizures occurred during periods of apparent well-being, nocturnal fasting, early in the morning with acetone breath and concomitant lactic acidosis on blood gas analysis. Epilepsy has not been described in GSD XII to date. However, the prevalent involvement of the CNS can be explained by the observation that the Aldolase A is the isoform most abundantly expressed in human brain [4] .Moreover, mild intellectual disabilities are described [6, 7, 9, 15] unveiling a chronic subtle neuronal involvement in some cases.

Mamoune et al. in 2014 described a similar Aldolase A pathogenic variant affecting 3 siblings with episodic rhabdomyolysis triggered by febrile illnesses. They showed that the underlying mechanism involved an exacerbation of aldolase A deficiency at high temperatures but that thermolability is very variable and tissue specific [15]. This is congruent with the clinical phenotype of our patient. In fact the crises were never triggered by fever.

Another previously described important feature of Aldolase A deficiency is hemolytic anemia. In our case, neither the proband nor the brother ever presented documented haemolytic anemia. The patient presented only mild normocytic anemia with hemolysis indexes within normal range, and he never required hemotransfusions or iron therapy, except at 2 years of age, but not surely related to the disease. Another discrepancy with other reports is the presence of lactic acidosis during metabolic decompensation in our patient. However, this feature might be explained by the glycolytic disruption of the disease.

To our knowledge, there is no effective therapy reported in the literature for patients affected by Aldolase A deficiency, besides one report showing that arginine supplementation could reduce rhabdomyolysis in vitro [15].

Our patient seemed to benefit from anti-epileptic therapy and avoiding of fasting by nocturnal corn starch, at beginning. Subsequently, with the aim to reduce glycogen storage in the muscle and provide alternative pathways in energy metabolism, independent from glycogen breakdown, a ketogenic diet was proposed to the patient. Ketogenic diet is a special high fat, carbohydrate-restricted diet that mimics the metabolic effect of fasting in order that ketone bodies become a significant energy source for extrahepatic tissues. This nutritional therapy has raised a lot of interest in refractory epilepsy, as well as amyotrophic lateral sclerosis, Alzheimer and Parkinson’s disease, and some mitochondriopathies [16].

There are previous reports on the use of ketogenic diet in patients affected by other types of muscular GSD, as type III, V, and VII. More specifically a 2-mo-old infant presenting with a familial form of GSD III complicated with cardiomyopathy was experimentally treated with a combination synthetic ketone bodies (D,L-3-OH butyrate) as an alternative energy source and 2:1 ketogenic diet in order to reduce glycogen accumulation. This led to an improvement of cardiomyopathy and to a normalization of liver size, with normal growth and no adverse effects reported [10]. In another report, 2 adult patients with GSD IIIa were treated with a ketogenic diet which led to a reduction of CK and a stabilization of blood glucose. Furthermore, it had a positive effect on progression of the disease, improving cardiomyopathy [11].

Similarly anecdotal reports in patients with GSD V and GSD VII showed an improvement in aerobic power and activity tolerance when patients were in a fasted state rather that in a fed state, this suggesting that alternative fuel substrates, such as ketone bodies, could potentially alleviate muscle symptoms [12]. Løkken et al. conducted an open-label pilot study, demonstrating how ketogenic diet improved fat oxidation rate, exercise capacity and subjective symptoms [13].

Our patient showed an important improvement of alert state and asthenia. He did not present new metabolic decompensations nor epileptic crises in five months of follow-up.

In conclusion, we described a novel phenotype of GSD XII, characterized by seizures, rhabdomyolysis, and lactic acidosis, without hemolytic anemia, in the absence of triggers as fever or exercise. Providing an alternative energy fuel to glucose for muscles and neurons, ketogenic diet resulted effective and safe in preventing metabolic decompensation. Therefore, GSD type XII might be considered a novel indication for ketogenic diet. A longer follow-up is necessary to verify our preliminary findings.

## Data Availability

The datasets used and/or analyzed during the current study are available from the corresponding author on reasonable request.

## Abbreviations

GSD: Glycogen storage disease
ALDOA: Aldolase A
